# Identification of metabolic pathways modulated by GAM and NGAM in the inhibition of *Staphylococcus aureus* biofilm formation

**DOI:** 10.3389/fmicb.2025.1689343

**Published:** 2025-11-06

**Authors:** Amirmohammad Afsharnia, Arjen Nauta, Andre Groeneveld, Blanca Fernandez-Ciruelos, Mostafa Asadpoor, Gert Folkerts, Saskia Braber, Marc Wösten

**Affiliations:** 1 Division of Pharmacology, Faculty of Science, Utrecht Institute for Pharmaceutical Sciences, Utrecht University, Utrecht, Netherlands; 2 Friesland Campina, Amersfoort, Netherlands; 3 Division of Infectious Diseases and Immunology, Department of Biomolecular Health Sciences, Faculty of Veterinary Medicine, Utrecht University, Utrecht, Netherlands

**Keywords:** arginine biosynthesis, biofilm metabolism, glucosamine (GAM), multidrug-resistant bacteria, N-acetylglucosamine (NGAM), RNA-seq, *Staphylococcus aureus*, TCA cycle

## Abstract

The prevalence of antibiotic-resistant bacterial strains, particularly *Staphylococcus aureus*, poses a significant threat to global health. The ability of *S. aureus* to form biofilms reduces the efficacy of antibiotics. Therefore, the need for innovative anti-biofilm strategies to improve the efficacy of antibiotic therapy is crucial, particularly when biofilms cause treatment failure. In this study, we investigated the effects of glucosamine (GAM) and its acetylated derivative, N-acetylglucosamine (NGAM), on the biofilm formation of the multidrug-resistant *S. aureus* strain Wood 46. The minimum biofilm inhibitory concentration (MBIC) assay was used to evaluate the inhibition of biofilm formation. The results indicated that 2–8% of GAM significantly inhibited *S. aureus* biofilm formation. However, only a high concentration of NGAM (8%) showed partial inhibition of biofilm formation. The RNA sequencing analysis of the treated biofilms indicated that, compared to NGAM, GAM leads to a more pronounced downregulation of *S. aureus* adhesion genes (*eno, ebps,* and *sraP*) and genes involved in arginine biosynthesis and tricarboxylic acid (TCA) pathways, which are essential for biofilm proteinaceous structure. The decreased pH in the biofilm environment treated with higher GAM concentrations supports its observed anti-biofilm activity and is likely linked to impaired pH homeostasis resulting from the downregulation of *ureABC* genes and disruption of urea metabolism, a process interconnected with arginine biosynthesis. In conclusion, unlike its acetylated form (NGAM), GAM is a potent anti-biofilm agent that effectively inhibits the biofilm formation of *S. aureus* Wood 46 and significantly alters the gene expression profile associated with biofilm formation.

## 1 Introduction

**Figure 1:**
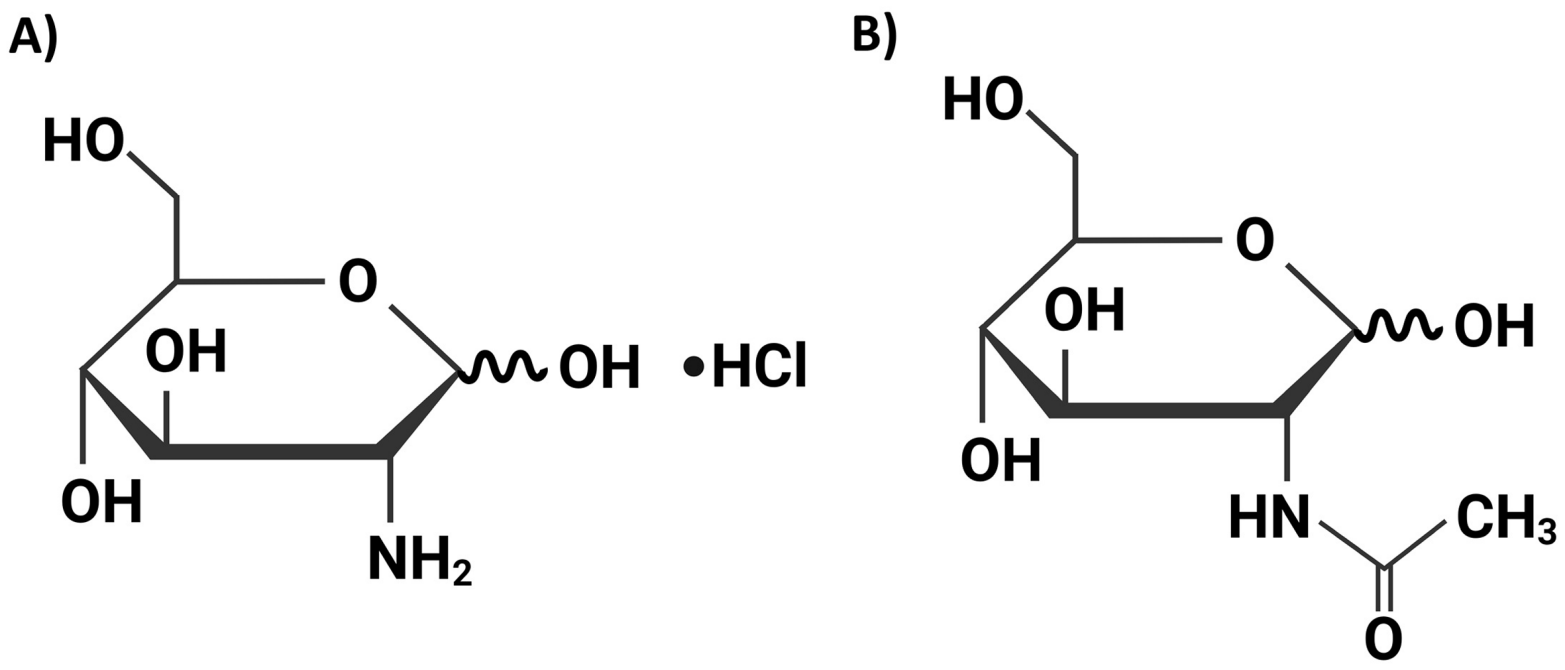
The chemical structures of D-(+)-glucosamine hydrochloride (GAM) **(A)** and N-acetyl-D-glucosamine (NGAM) **(B)**. These structures were drawn using the web-based BioRender software.

The increasing prevalence of antibiotic-resistant bacterial strains is one of the major global public health threats. It was estimated that antibiotic-resistant bacterial infections were directly responsible for more than one million deaths in 2019, which could increase drastically to 50 million by 2050 without well-adapted preventive measures (O’Neill, 2014). One of the primary multidrug-resistant microorganisms is *Staphylococcus aureus* (*S. aureus*) (Pajohesh et al., 2022; Lowy, 1998). *S. aureus* is a Gram-positive opportunistic bacterial pathogen commonly found as part of the skin and nasal cavity flora. Under certain circumstances, *S. aureus* can become pathogenic, causing a wide range of health issues in humans and animals (Lowy, 1998; Petersson-Wolfe et al., 2010). Bacteria such as *S. aureus* continuously attempt to evade the host immune system or antimicrobial agents by using different mechanisms. *S. aureus* strains are categorized as methicillin-resistant *S. aureus* (MRSA) or methicillin-sensitive *S. aureus* (MSSA) strains (Chambers and DeLeo, 2009). Biofilm formation is one of the key mechanisms of *S. aureus* bacterial resistance against antimicrobial agents (Hall-Stoodley et al., 2004; Prinzi and Rohde, 2023). *S. aureus* biofilm formation is initiated after planktonic bacteria adhere to a biotic or abiotic surface, followed by the secretion of an extracellular matrix (ECM) (Moormeier and Bayles, 2017). The biofilm structure varies from a more proteinaceous composition in MRSA strains to a polysaccharide composition in MSSA strains (Pozzi et al., 2012). A biofilm forms through five different stages, each regulated by several encoding genes (Moormeier and Bayles, 2017; Wu et al., 2024). Disturbing any of these stages could inhibit biofilm formation or facilitate its dispersal (Roy et al., 2018). To overcome antibiotic-resistant *S. aureus* strains and biofilm formation as protective mechanisms for bacteria, novel compounds should be considered in the search for alternative treatments. Naturally accessible compounds, such as carbohydrates with various molecular structures, might be significant therapeutic alternatives (Ritter and Wong, 2001; Ye and Chen, 2022). Glucosamine (GAM) and its acetylated derivative, N-acetylglucosamine (NGAM), are amino-monosaccharides (Figure 1) with broad-spectrum applications in the food and pharmaceutical industries (Dalirfardouei et al., 2016; Chen et al., 2010; Liu et al., 2013; Noack et al., 1994). GAM significantly enhances the potential antibacterial properties of substances such as gold, silver, and copper nanoparticles against *S. aureus* (Veerapandian et al., 2010; Yang et al., 2018; Chudobova et al., 2015). Increased anti-staphylococcal activity was also observed when GAM was added to an oligochitosan solution (Blagodatskikh et al., 2013). The improved efficacy against *S. aureus* is attributed to the increased interactions between these nanoparticles and the bacterial cell wall after the addition of GAM, allowing the compounds to penetrate the bacteria (Blagodatskikh et al., 2013). Importantly, chitosan oligosaccharides (COSs), which are composed of monomeric units of GAM and NGAM, have demonstrated potent anti-biofilm activity against *S. aureus*. COSs not only inhibit biofilm formation but also show synergistic effects with antibiotics such as clindamycin, enhancing their efficacy against *S. aureus* biofilms (Asadpoor et al., 2021). These findings suggest that the monomeric building blocks of COSs, GAM and NGAM, may themselves possess biofilm-inhibitory properties, warranting investigation into their individual effects.

To the best of our knowledge, no studies have investigated the impact of GAM on *S. aureus* biofilm formation. Given the critical role of bacterial biofilms in the prevalence of antibiotic-resistant strains, we investigated the effects of GAM and NGAM on *S. aureus* biofilm formation and their potential anti-biofilm mechanisms. These compounds differ subtly in chemical structure, particularly due to the presence of an acetyl group in NGAM (Figure 1). The biofilm assay illustrated complete inhibition of *S. aureus* biofilm formation at various concentrations of GAM, while only partial inhibition was observed at the highest concentration of NGAM. Transcriptomic analysis revealed that GAM inhibits *S. aureus* biofilm formation by suppressing genes involved in bacterial adhesion and inhibiting dominant metabolic pathways; however, this inhibitory effect was less pronounced with NGAM.

## 2 Materials and methods

### 2.1 Bacterial strain

*S. aureus* Wood 46 (ATCC 10832; de Vor et al., 2022), a MRSA strain, was generously provided by Prof. Dr. S. Rooijakkers (UMC, University Medical Center; Utrecht, Netherlands). The strain was cultured on sheep blood agar plates (Biotrading, Mijdrecht, Netherlands) at 37 °C.

### 2.2 GAM and NGAM

D-(+)-glucosamine hydrochloride (GAM) derived from *Aspergillus niger* and N-acetyl-D-glucosamine (NGAM) derived from *Paralithodes camtschaticus*, both with a purity ≥99%, were purchased from Sigma-Aldrich (St. Louis, MO, United States). The compounds were freshly prepared by dissolving them in tryptic soy broth (TSB); their pH was adjusted to ≈7.3; and they were finally passed through a 0.2 μm sterile filter (Minisart®, Sartorius, Göttingen, Germany). The chemical structures of GAM and NGAM are depicted in Figure 1.

### 2.3 Minimum biofilm inhibitory concentration (MBIC) assay

The minimum biofilm inhibitory concentration (MBIC) assay was conducted to evaluate the effect of GAM and NGAM on the biofilm formation of *S. aureus*. For this purpose, *S. aureus* was cultured in TSB for 24 h at 37 °C in a shaking incubator (160 rpm). After 24 h, the culture was diluted to achieve a starting optical density (OD_600nm_) of ~0.0005 for the MBIC assay. The MBIC assay, with or without GAM or NGAM at concentrations ranging from 8 to 0.25%, was performed in TSB supplemented with 0.5% glucose and 3% NaCl [biofilm media or BM (Kang et al., 2019)], resulting in a final volume of 200 μL in a 96-well F-bottom polystyrene microtiter plate.

The MBIC assay plate was covered with a sterile breathable cover (VWR International, Amsterdam, Netherlands) and incubated at 37 °C with 5% CO_2_ under static conditions for 24 h. After incubation, the supernatant was removed from the wells, and the biofilms were rinsed with 200 μL of phosphate-buffered saline (PBS). Then, the biofilms were fixed at 60 °C for 30 min and stained with 160 μL of 0.1% crystal violet (CV) for 5 min. The CV stain from the wells was carefully washed once with excess tap water, and the stained biofilms at the bottom of the wells were dissolved in 160 μL of 33% acetic acid. Finally, 100 μL of the dissolved biofilm was transferred to another 96-well plate, and the optical density at 595 nm was measured using a FLUOstar Omega microplate reader.

The MBIC of GAM and NGAM was statistically analyzed as previously described by Sun et al. (2016), based on the inhibition of bacterial biofilm growth exceeding 90% compared to the control with full biofilm growth (positive control).

### 2.4 Colony-forming unit (CFU) assay

The colony-forming unit (CFU) assay was performed on different biofilm conditions to measure the number of surviving bacteria in the biofilm of *S. aureus.* Briefly, after 24 h of the MBIC assay, the supernatant was removed and the biofilms were rinsed with 200 μL of PBS. Subsequently, 100 μL of PBS was added to each well, and the biofilms were scratched with sterile tips. Serial dilutions of the well content were cultured on sheep blood agar square Petri dishes and incubated at 37 °C. The number of *S. aureus* colonies was counted after 24 h, and the data are shown in Supplementary Figure S1.

### 2.5 RNA isolation from *S. aureus* biofilms

The culture media above the biofilm were first removed before total RNA isolation from the *S. aureus* biofilms. TRIzol™ LS Reagent (Invitrogen, United States) was used for total RNA isolation according to the manufacturer’s instructions. To ensure that the collected RNA samples yield good results, we measured the bacterial counts in both the supernatant and the biofilm. Due to the very low bacterial population in the supernatant, we decided to conduct RNA sequencing exclusively on the biofilm samples. The RNA yield was obtained by pooling eight different wells, and three independent biological replicates were collected for each condition for subsequent RNA sequencing.

### 2.6 RNA-sequencing (RNA-seq)

To achieve the best possible results, a quality control (QC) process was performed on the isolated RNA samples. All samples with an RNA quantity of ≥500 ng and ODs of A260/230 and A260/280 ≥ 2 passed the QC. Subsequently, to ensure the absence of DNA contamination, the extracted RNA samples were subjected to treatment with RNase-free DNase I (Thermo Fisher Scientific, Baltics UAB, Lithuania). The prepared RNA samples were delivered to Novogene (Novogene UK, Cambridge, United Kingdom) for RNA-sequencing. The RNA library was prepared through rRNA removal and cDNA reverse transcription. Illumina Novaseq6000 was used to sequence the RNA samples, and the NovaSeq PE150 strategy was then utilized to screen the expressed genes. Transcriptomic analysis was then performed to identify differentially expressed genes (DEGs). The log_2_(FPKM+1) scale was utilized to represent the expression levels of genes, which also indicates the fold change in the expression of each gene compared to the expression level in the reference (positive control). For this purpose, all expressed genes were classified based on the corrected *p*-value (−log10) into non-differentially expressed genes and differentially expressed genes. The expression levels of DEGs in the treated biofilm samples (GAM and NGAM) were compared to the positive control, and the fold change for each gene was determined. The fold change factor represents the difference in the expression of each specific gene in the treated biofilm samples compared to the positive control. The output data were displayed as negative and positive values, representing downregulated and upregulated genes, respectively. Through enrichment analysis of DEGs, the clustering of expressed genes associated with different pathways was investigated. There are different databases offering a wide range of pathways, such as biological pathways, genomes, and diseases. In the present study, the Kyoto Encyclopedia of Genes and Genomes (KEGG) database was used to identify pathways related to the clustering of DEGs. Subsequently, clusterProfiler software (version 4.0) was used for enriching DEGs (Wu et al., 2021). Different functional pathways were found by grouping upregulated or downregulated DEGs and differentiated based on the number of involved genes and the adjusted *p*-value (−log10). Hierarchical clustering analysis was also conducted to categorize genes with similar expression patterns into different clusters, which were then illustrated in a heatmap.

### 2.7 pH evaluation of the supernatant of *S. aureus* biofilms

To measure the pH of the biofilm supernatant, the upper media from similarly treated wells were carefully collected and pooled in a 50 mL sterile tube in order to obtain a sufficient volume for accurate measurement using a pH meter probe.

### 2.8 Statistical analysis

The data were presented as mean ± SEM from a minimum of three separate experiments (*n* = 3), each conducted in triplicate (three wells per group). The results were statistically analyzed using GraphPad Prism 9.0 software (GraphPad, San Diego, CA, United States). Statistical significance was assessed using one-way ANOVA, followed by the Bonferroni *post hoc* test, with *p*-values less than 0.05 considered statistically significant.

## 3 Results

### 3.1 GAM and NGAM demonstrate distinct effects on the biofilm formation of *S. aureus*

**Figure 2:**
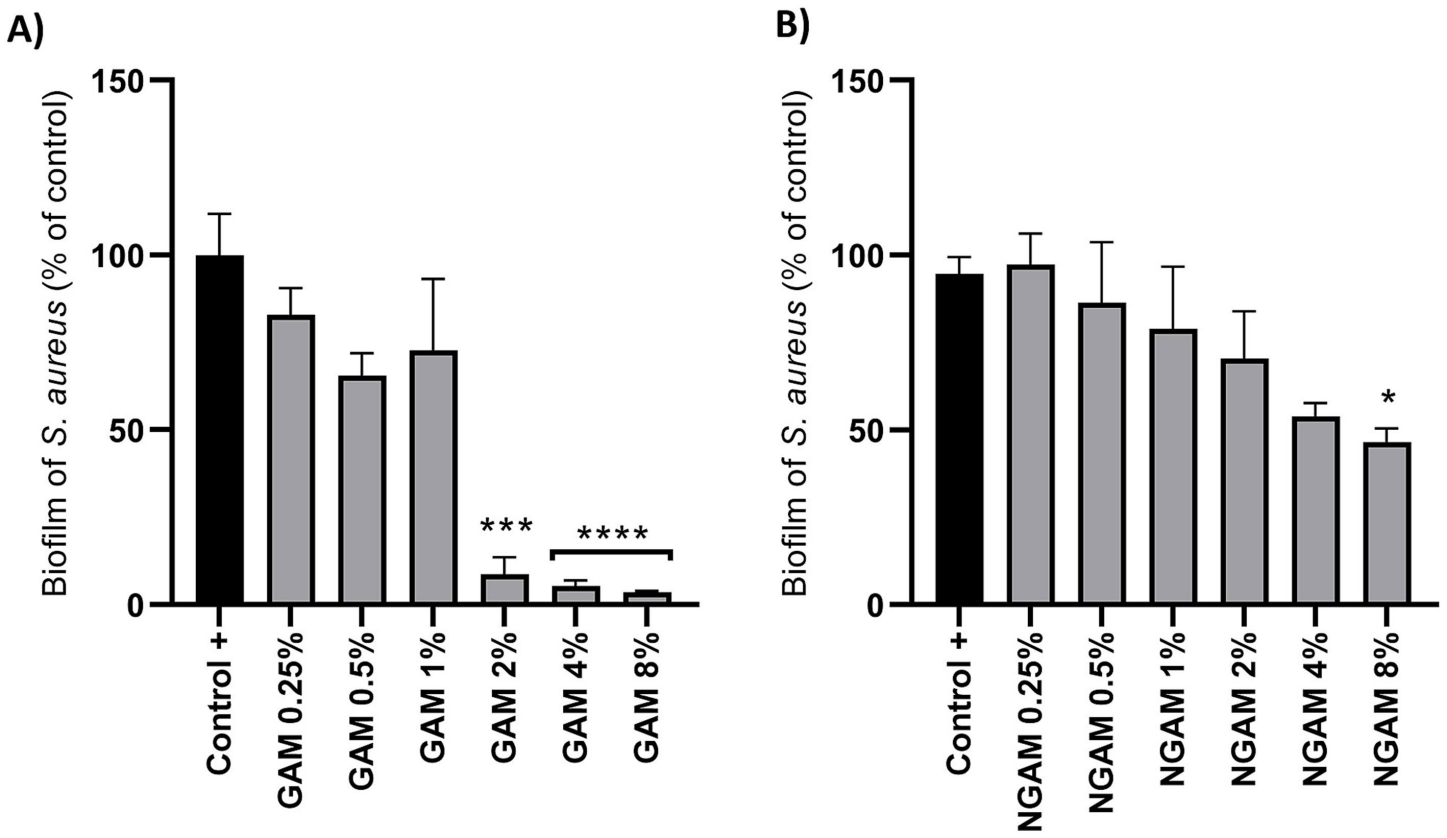
**(A)** The activity of GAM and **(B)** NGAM against the biofilm formation of the *S. aureus* Wood 46 strain. Two-fold serial dilutions of GAM and NGAM in TSB were exposed to the *S. aureus* biofilm for 24 h. The positive control (Control+) represents the full biofilm formation of *S. aureus* Wood 46 in TSB media. The data are presented as mean ± SEM, expressed as a percentage of full biofilm formation (Control+). The data were obtained from three different biological and technical replicates. The values for each condition were statistically compared to the positive control using one-way ANOVA (^*^p ≤ 0.05, ^***^p ≤ 0.001, and ^****^p ≤ 0.0001). GAM, glucosamine; NGAM, N-acetylglucosamine.

The inhibitory activity of GAM and NGAM against *S. aureus* biofilm formation was compared using the CV staining protocol. As depicted in Figure 2, the serially diluted concentrations of both compounds, ranging from 0.25 to 8%, were applied to the biofilms of *S. aureus*. Both compounds showed inhibitory effects against biofilm formation. However, the extent of inhibition varied between the two compounds. Although a concentration-dependent biofilm reduction was observed with NGAM, only 8% NGAM significantly inhibited biofilm formation. The inhibition of biofilm formation reached 47% with 8% NGAM and 54% with 4% NGAM compared to full biofilm formation (the positive control, Figure 2B). In contrast, GAM demonstrated a more potent inhibitory effect, leading to drastic biofilm inhibition. Among all the tested concentrations of GAM and NGAM, GAM at concentrations ≥2% can be considered the MBIC against *S. aureus*, achieving over 90% inhibition of biofilm formation (Figure 2A). The CFU assay showed a significantly lower number of live *S. aureus* bacteria in all GAM-treated biofilms compared to the NGAM-treated biofilms (Supplementary Figure S1).

### 3.2 GAM and NGAM alter the gene expression profile of the *S. aureus* biofilm

**Figure 3:**
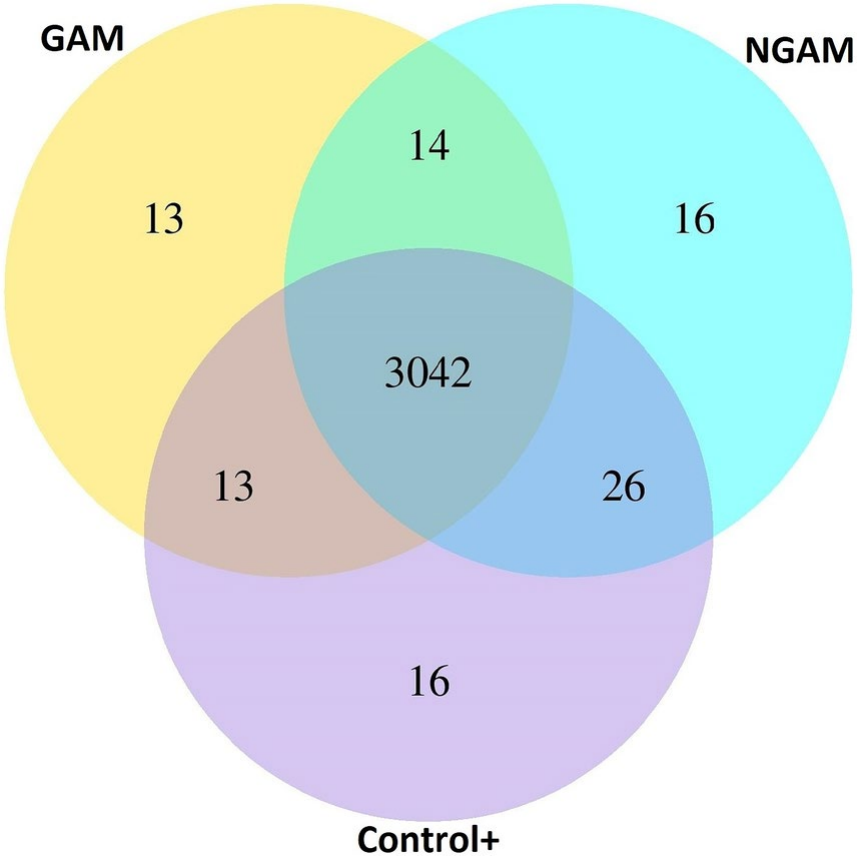
Venn diagram showing overlapping and unique differentially expressed genes (DEGs) among the GAM-treated, NGAM-treated, and untreated *S. aureus* biofilms (24 h). Control+ represents the full biofilm formation of *S. aureus* in TSB (positive control). GAM and NGAM represent the biofilms treated with GAM and the biofilms treated with NGAM, respectively.

To gain insights into the mechanism underlying the differential effects of GAM and NGAM on *S. aureus* biofilm formation, the transcriptomic profiling of the treated and untreated biofilms was compared. Due to the low remaining biofilm biomass under inhibited conditions and to obtain sufficient RNA, *S. aureus* biofilms exposed to 0.5% concentrations of GAM and NGAM were selected for RNA isolation. A Venn diagram (Figure 3) was generated to depict the co-expressed and unique differentially expressed genes (DEGs) among the GAM-treated, NGAM-treated, and untreated *S. aureus* biofilms. Of all 3,140 expressed genes, 3,042 genes were commonly expressed in the biofilms treated with GAM and NGAM, as well as in the untreated biofilms (Supplementary Tables S1,S2). However, only a limited number of genes were uniquely expressed under each biofilm condition (13 for GAM, 16 for NGAM, and 16 for control).

**Figure 4:**
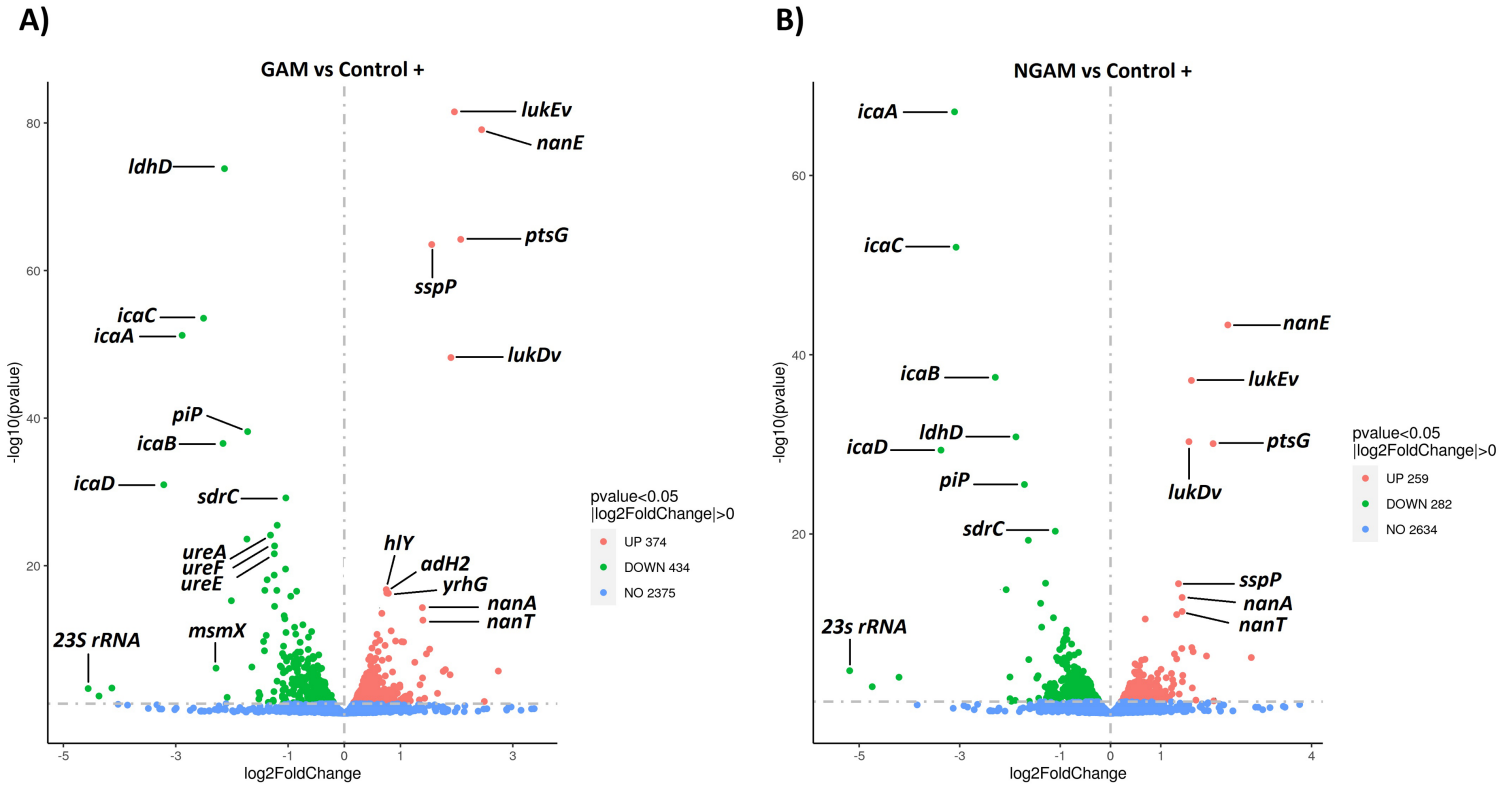
Volcano plots visualizing the distribution of differentially expressed genes (DEGs) in the *S. aureus* Wood 46 strain biofilms exposed to **(A)** GAM and **(B)** NGAM, compared to untreated biofilms (positive control or Control+). Gene expression was statistically analyzed, and genes with a corrected *p*-value of *≤*0.05 were considered DEGs. The horizontal axis represents the fold change in gene expression, while the vertical axis represents the −log10 corrected *p*-value. Smaller *p*-values are indicated higher on the y-axis. Each point on the plot represents a specific gene. Blue dots represent genes with no significant differential expression, red dots represent upregulated DEGs, and green dots represent downregulated DEGs. Due to the proximity of gene expression values, only 12 highly significant genes, based on *p*-values and/or fold changes, are labeled on the plots. The data for each sample were derived from three biological and technical replicates. GAM, glucosamine; NGAM, N-acetylglucosamine.

Subsequently, the classified genes based on the *p*-value (≤0.05) and fold change factors were illustrated in volcano plots (Figures 4A,B). In the *S. aureus* biofilm treated with GAM and NGAM, a total of 808 and 541 DEGs were identified, respectively. The highly downregulated DEGs, such as *ldhD, icaABCD, ureAEF, sdrC,* and *23S rRNA,* were similar in both GAM- and NGAM-treated biofilms, although the fold change in gene expression was slightly different. Similarly, the highly upregulated DEGs, such as *nanAET, lukEv, lukDv, ptsG, and sspP,* were similarly expressed in both GAM- and NGAM-treated biofilms, with only small differences in the fold change of gene expression.

### 3.3 Several upregulated and downregulated biological pathways are detected through the clustering of differentially expressed genes and KEGG enrichment analysis

**Figure 5:**
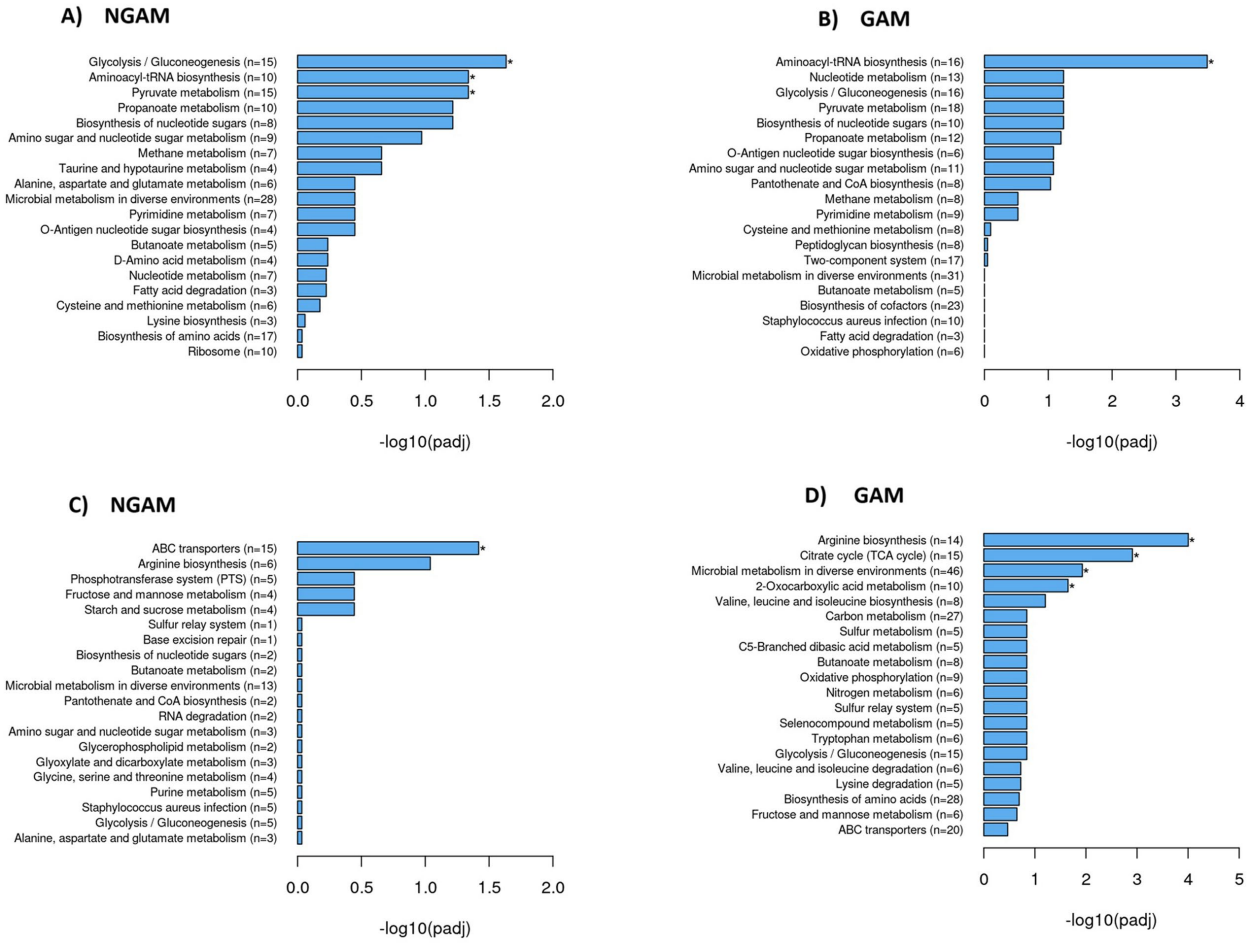
Significantly upregulated and downregulated KEGG pathways in the *S. aureus* Wood 46 biofilms treated with GAM or NGAM. The y-axis shows the pathway name and the number of involved genes (rich factor) per pathway (*n* = 3), while the x-axis represents the −log10 adjusted *p*-value after multiple hypothesis testing, shown as histograms. A star on the bar indicates pathways with statistically significant differences in the rich factor. The rich factor is defined as the ratio of DEG counts to the pathway’s annotated gene counts, reflecting the degree of enrichment. Panels **(A)** and **(B)** show histograms of upregulated KEGG pathways in the biofilms treated with NGAM and GAM, while panels **(C)** and **(D)** display the corresponding data for downregulated pathways in the biofilms treated with NGAM and GAM, respectively.

The KEGG database was used to compare the investigated DEGs against the whole genome background of the *S. aureus* Wood 46 strain, identifying significantly enriched metabolic pathways. The results were visualized using histograms, as depicted in Figure 5. Glycolysis/gluconeogenesis, aminoacyl-tRNA biosynthesis, and pyruvate metabolism pathways were found to be significantly upregulated in the biofilm treated with NGAM (Figure 5A). In contrast, only the aminoacyl-tRNA biosynthesis pathway was significantly upregulated in the biofilm treated with GAM (Figure 5B). Although only the ABC transporter pathway was significantly downregulated in the NGAM-treated biofilms (Figure 5C), four different pathways, including arginine biosynthesis, citrate cycle, microbial metabolism in diverse environments, and 2-oxocarboxylic acid metabolism, were significantly downregulated in the biofilms treated with GAM (Figure 5D).

**Figure 6:**
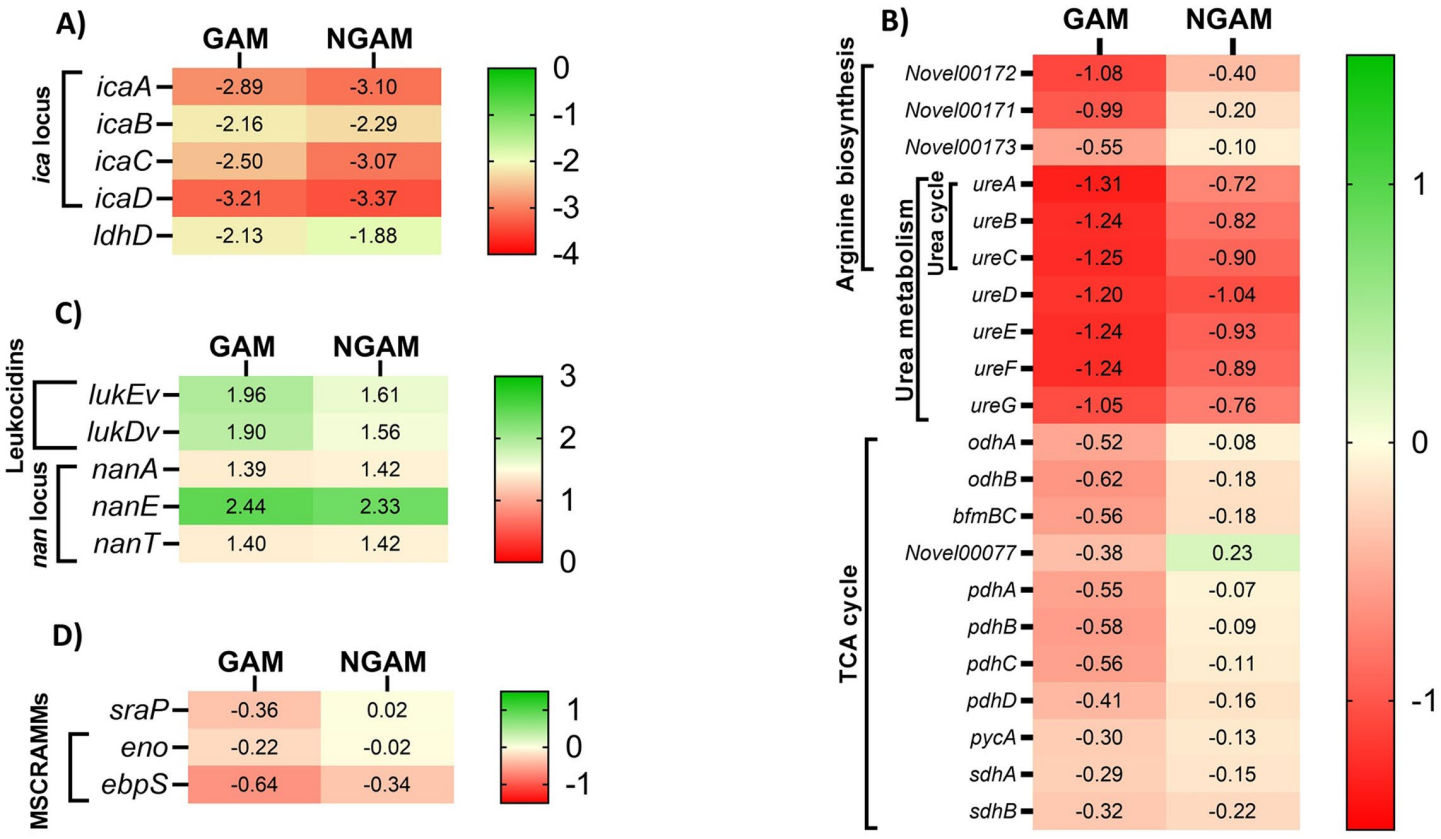
The heatmaps display cluster analysis of gene expression levels in the *S. aureus* Wood 46 biofilms treated with GAM (left columns) or NGAM (right columns). **(A)** The highly downregulated *ldhD* and *ica* locus genes, **(B)** genes involved in the TCA cycle, arginine biosynthesis KEGG pathways, and urea metabolism, **(C)** highly upregulated genes including leukocidins and *nan* locus genes, and **(D)** genes involved in MSCRAMMs, such as the *sraP* gene, were selected for generating the heatmaps. The color ranges from green to red, representing fold changes from high to low, respectively. The values in each cell indicate the fold change in the expression levels of the related genes under each condition, varying from positive values (upregulated) to negative values (downregulated).

Given the stronger anti-biofilm activity of GAM, as illustrated in Figure 2, we aimed to determine which genes involved in biofilm formation are more significantly affected by GAM compared to NGAM. To accomplish this, we focused on genes specifically associated with the two most downregulated pathways: the tricarboxylic acid (TCA) cycle and arginine biosynthesis. Additionally, differentially expressed genes (DEGs) known to play a role in biofilm formation were included. Heatmaps depicting these genes were generated, as illustrated in Figure 6. Polysaccharide intercellular adhesin (PIA)-encoding genes (*icaABCD*) and *ldhD* were among the most downregulated genes in both GAM- and NGAM-treated biofilms, as shown in Figure 6A. On the other hand, *S. aureus* leukocidin-encoding genes (*lukEv and lukDv*) and *nan* locus genes (*nanAET*) were highly upregulated under both GAM and NGAM conditions, as displayed in Figure 6C. The transcription of the majority of the genes involved in the TCA cycle, arginine biosynthesis, and urea metabolic pathways was lower in the biofilm treated with GAM than in the biofilm treated with NGAM (Figure 6B). In particular, there was a difference in the expression of the downregulated *ureA-C* and *pdhA-D* genes between the biofilms treated with GAM and NGAM. In addition, genes involved in the initial stages of prokaryotic *S. aureus* adhesion and colonization, such as *sraP*, as well as microbial surface components recognizing adhesive matrix molecules (MSCRAMMs), were mostly downregulated by GAM (Figure 6D).

### 3.4 pH of the *S. aureus* biofilm environment changes as the biofilm is inhibited by GAM

**Figure 7:**
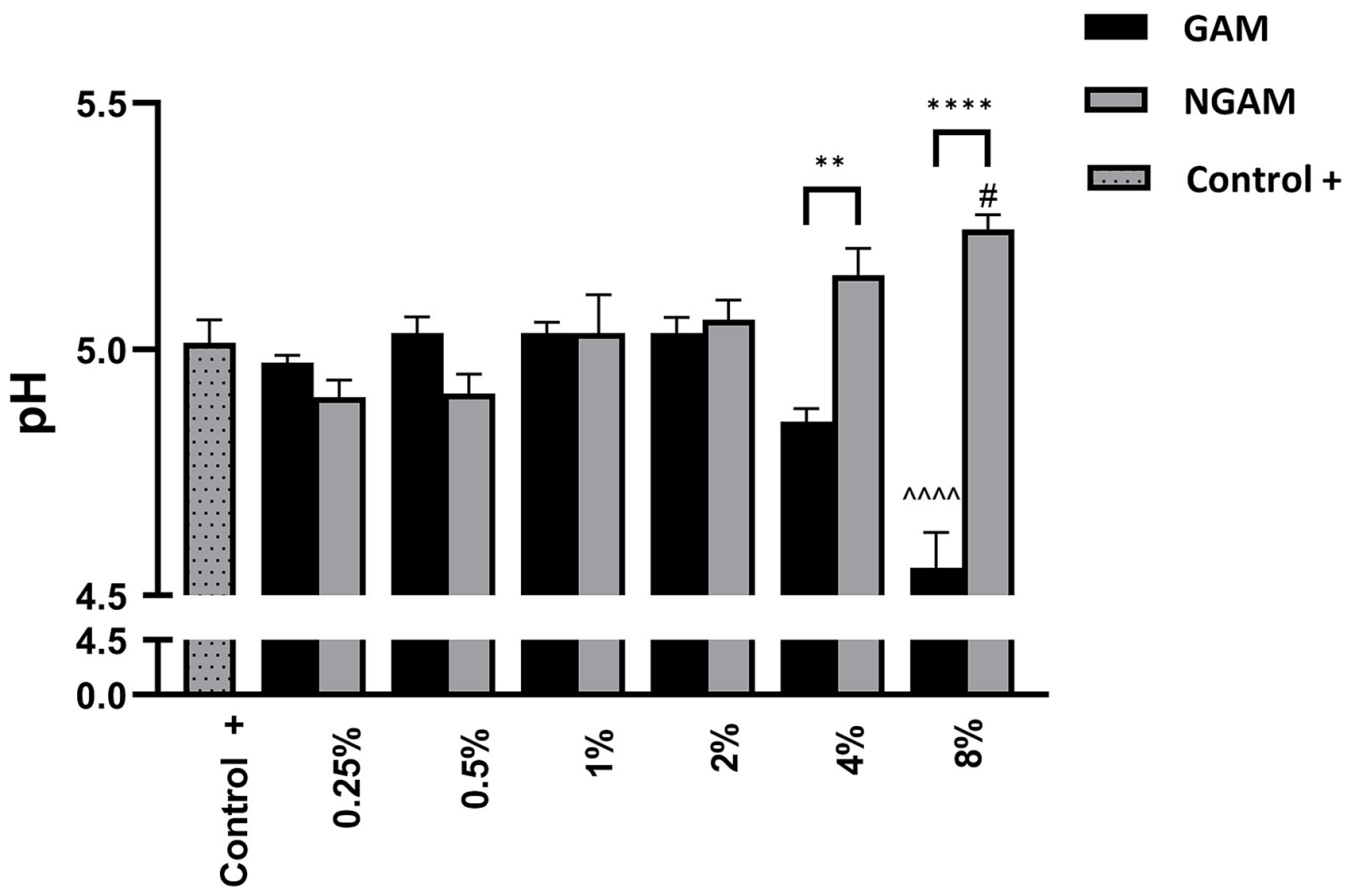
The pH of the supernatant from the *S. aureus* Wood 46 biofilms following supplementation with GAM and NGAM. The pH values are presented on the y-axis, while the experimental conditions are displayed on the x-axis. The positive controls (Control+), representing the pH of the supernatants from the fully developed biofilms, are shown in dotted columns. Data were analyzed using one-way ANOVA to compare the supernatants across similar treatment conditions (^**^p ≤ 0.01, ^****^p ≤ 0.0001). In addition, the pH of each treated biofilm supernatant was compared to its respective positive control (Control+) using one-way ANOVA: 8% GAM versus GAM control (^^^^^^*p* ≤ 0.0001) and 8% NGAM versus NGAM control (^#^*p* ≤ 0.05).

To assess the metabolic impact of GAM and NGAM on *S. aureus* biofilm formation, changes in the pH of the supernatant from the biofilm growth environment were monitored, as shown in Figure 7. The supernatant of the untreated *S. aureus* biofilm (positive control) exhibited a moderately acidic pH (≈5.0). Treatments with lower concentrations of GAM and NGAM (0.25–2%), which exhibited no or minimal anti-biofilm activity, resulted in supernatant pH values similar to those of the controls. However, treatments with higher concentrations (4 and 8%) revealed opposing pH trends between GAM and NGAM. The supernatants from biofilms treated with 4 and 8% NGAM showed the highest pH values among all tested conditions (≈5.15 and ≈5.24, respectively), whereas treatment with 4 and 8% GAM resulted in the lowest pH values (≈4.85 and ≈4.56, respectively).

## 4 Discussion

*S. aureus* is recognized as a major antibiotic-resistant bacterium that causes complex infections in humans and animals (Tacconelli, 2017; Rao et al., 2022). Almost all *S. aureus* strains can form a biofilm, which is one of the key mechanisms of bacterial protection against antibiotics (Hall-Stoodley et al., 2004). Considering the major role of the biofilm in protecting *S. aureus* under stressful conditions, we examined the effects of the amino-monosaccharides GAM and its acetylated form NGAM on *S. aureus* biofilm formation and investigated their potential anti-biofilm mechanisms.

A strong (>90%) and significant inhibition of *S. aureus* Wood 46 biofilm formation was observed after treatment with 2 to 8% GAM, while 8% NGAM could only inhibit biofilm formation by 47%. Similar results have been obtained using polysaccharide chitosan, which typically contains 60–90% GAM depending on the degree of deacetylation, with the remaining portion being NGAM (Asli et al., 2017; Felipe et al., 2019; Lopes et al., 2020). However, the reported inhibitory effect of chitosan on biofilm formation was less pronounced compared to that of GAM. NGAM is not only a main component of the bacterial cell wall but also forms polymers (PNAG or PIA) that are an important part of the biofilm matrix and play an important role in the adhesion of *S. aureus* to solid surfaces during biofilm formation. This likely explains why NGAM interferes less with biofilm formation than GAM (Lin et al., 2015; Yeswanth et al., 2013).

The RNA-sequencing analysis performed on the *S. aureus* biofilm exposed to GAM and NGAM displayed an altered biofilm gene expression profile induced by both amino-monosaccharides. Among the investigated DEGs, all genes encoding *S. aureus* PIA-producing enzymes (*icaABCD*) were highly downregulated by both GAM and NGAM. The presence of ica enzymes is crucial for cell–cell adhesion and subsequently for forming biofilms by *S. aureus* planktonic bacteria (Cramton et al., 1999; Diemond-Hernández et al., 2010). Other studies using anti-biofilm compounds, such as gallic acid or manuka honey, have shown that downregulation of the *ica* genes, particularly *icaA and icaD,* is crucial for the reduction of *S. aureus* biofilms (Liu et al., 2017; Kot et al., 2020). Although NGAM showed no significant inhibitory effect on *S. aureus* biofilm formation, the *icaABCD* genes were found to be nearly as downregulated as those associated with GAM. Therefore, downregulation of the *ica* gene family is unlikely to be the main mechanism behind the anti-biofilm properties of GAM.

**Figure 8:**
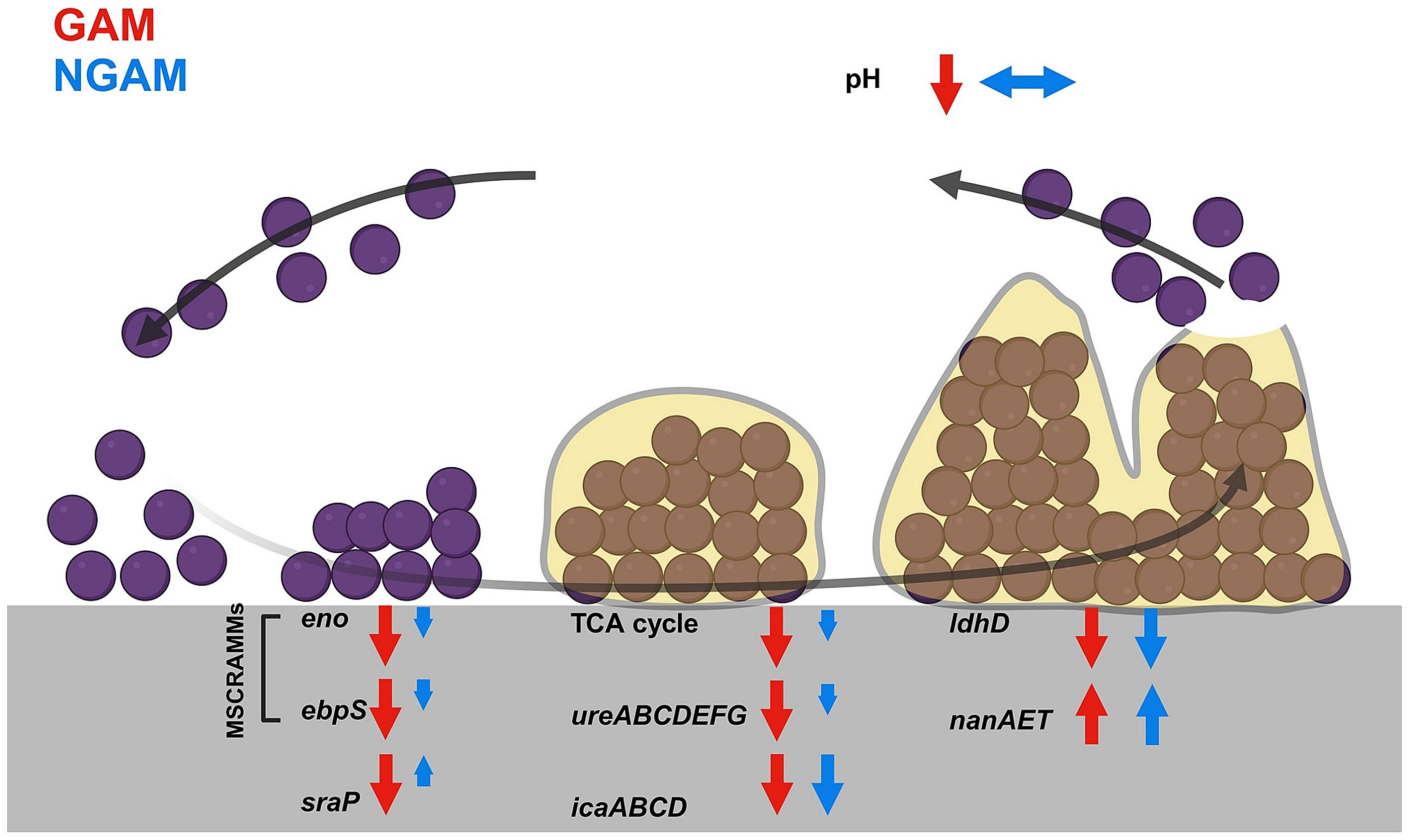
This figure shows the main findings of the study. The biofilm of *S. aureus* develops through five different stages. These stages, from left to right, include prokaryotic *S. aureus* attachment, multiplication, exodus, maturation, and dispersal. The most significant changes under GAM and NGAM conditions are indicated at each stage of biofilm formation. The modulations induced by GAM are depicted with red arrows, while those under NGAM conditions are shown with blue arrows. Modulated genes, loci, or metabolic pathways are indicated with upward arrows for upregulation and downward arrows for downregulation. The length of the arrows implies the relative strength of modulation under GAM and NGAM conditions.

Treating the *S. aureus* biofilm with GAM and NGAM resulted in the high expression of many genes with roughly similar expression levels, particularly in the highly upregulated genes such as *lukDv* and *lukEv*, as shown in Figures 4, 8. The *lukED* genes encode *Staphylococcal* strain-specific toxins with cytolytic functions, which are secreted as virulence factors for pathogenesis purposes (Vasquez et al., 2020). Furthermore, we observed a similar upregulation level in some of the *nan* locus (*nanA, nanE, nanT*) genes under both GAM and NGAM conditions, which are part of carbon metabolism in the bacteria (Olson et al., 2013). The *nan* genes facilitate the uptake and catabolism of sialic acid, allowing *S. aureus* to adapt to environments rich in this nutrient. Since sialic acid, which is converted into pyruvate in the glycolytic pathway, contributes to the structural integrity and stability of biofilms, upregulation of the *nan* genes may destabilize the biofilm structure (Olson et al., 2013).

However, the adaptation of *S. aureus* biofilms to diverse environments is achieved through several biological pathways (Malviya et al., 2023). Both GAM, and to a lesser extent NGAM, downregulated TCA cycle genes encoding the pyruvate dehydrogenase (PDH) complex (*pdhABCD*), succinate dehydrogenase (*sdhAB*), and oxoglutarate dehydrogenase (*odhAB*) compared to the untreated biofilm (Figure 6A). The TCA cycle plays a central role in *S. aureus* biofilm metabolism and ATP production, especially under conditions of oxygen and nutrient shortages (Gaupp et al., 2010). Within biofilms, oxygen availability is limited, creating microaerophilic to anaerobic niches. Under such conditions, *S. aureus* upregulates genes in glycolysis, fermentation, and anaerobic respiration while repressing genes in the TCA cycle (Fuchs et al., 2007; Cramton et al., 2001). However, other studies have shown that, in certain biofilm regions, the enzymes and corresponding genes of the TCA cycle are upregulated compared to planktonic growth (Resch et al., 2005; Resch et al., 2006), indicating metabolic heterogeneity and suggesting that GAM, in particular, facilitates the transition from a biofilm to a planktonic growth phase (Resch et al., 2005; Resch et al., 2006).

In addition to TCA metabolism, the arginine biosynthesis pathway was found to be downregulated in both GAM- and NGAM-treated *S. aureus* biofilms. Our data indicate a more pronounced downregulation of the *ureABC* genes in the *S. aureus* biofilm treated with GAM compared to NGAM (Figures 6, 8). The arginine biosynthesis pathway enables the bacterium to process different biological needs, such as protein synthesis and biofilm formation (Manna et al., 2022; Zhu et al., 2007). Arginine biosynthesis might be one of the protein synthesis pathways responsible for the formation of the proteinaceous biofilm structure of antibiotic-resistant *S. aureus* strains, such as Wood 46 (Pozzi et al., 2012). The biological processes involved in *S. aureus* biofilm formation can be severely disrupted when arginine biosynthesis is downregulated, as observed in the enrichment analysis of GAM (Manna et al., 2022). Disrupting arginine biosynthesis is important for inhibiting biofilm persistence; however, it leads to increased antibiotic tolerance in *S. aureus* biofilms (Freiberg et al., 2024). The urease enzyme complex, encoded by *ureABC*, plays a pivotal role in neutralizing acidic conditions within the biofilm by hydrolyzing urea into ammonia and carbon dioxide, maintaining pH homeostasis, and promoting biofilm persistence (Zhou et al., 2019; Zhou and Fey, 2020). Disruption of urease activity can compromise acid resistance, leading to biofilm destabilization (Zhu et al., 2007). In addition, the biofilms of *S. aureus* exhibited expression of both the urease genes (*ureABC*) and the lactate dehydrogenase (LDH)-encoding gene (*ldhD*), which together facilitate pH regulation under weak acidic stress during biofilm growth (Figure 6) (Zhou et al., 2019). This acidic stress arises from pyruvate produced through anaerobic glycolysis, most of which is converted to lactic acid by LDH (Olson et al., 2013). These findings suggest that the superior anti-biofilm activity of GAM may be linked to its ability to impair pH regulation mechanisms, particularly through urease inhibition. In agreement with the downregulation of the urease genes, we observed that the pH of the biofilm cultures decreased significantly after treatment with 2–8% GAM but not after treatment with NGAM (Figure 7). The more pronounced downregulation of the *ureABC* genes involved in *S. aureus* biofilm arginine metabolism by GAM may explain its superior anti-biofilm activity compared to NGAM. These findings suggest that GAM inhibits both aerobic (TCA) and anaerobic (arginine biosynthesis) energy metabolism pathways of the *S. aureus* biofilm, highlighting its potential as a more effective agent for inhibiting *S. aureus* biofilm formation.

Then, we investigated the expression of several key regulatory genes involved in biofilm formation (Wu et al., 2024), which may provide insights into the observed effects of GAM and NGAM. The encoding genes of the elastin-binding protein (*ebpS*), laminin-binding protein (*eno*), and serine-rich adhesin for binding to platelets (*sraP*) were more downregulated by GAM compared to NGAM (Figure 6D). The *eno* and *ebpS* genes encode MSCRAMMs, which facilitate bacterial adhesion to different surfaces and play a major role in biofilm formation (Foster, 2019; Zuniga et al., 2015; Nemati et al., 2009). On the other hand, the *sraP* gene encodes a surface glycoprotein involved in *S. aureus* adhesion to human platelets and bacterial aggregation. However, its expression has been reported to vary among different *Staphylococcus* strains (Yang et al., 2014; Sanchez et al., 2010). The expression of *eno* and *ebpS* genes is reported to be higher in MRSA strains that produce stronger biofilms (Kot et al., 2018). The expression levels of the MSCRAMM-encoding genes (*eno* and *ebpS*) and the biofilm surface molecule (*sraP*) are lower in GAM-treated biofilms than in NGAM-treated biofilms. This suggests that the anti-biofilm properties of GAM are also due to its significant anti-adhesive activity.

In conclusion, both GAM and NGAM are capable of reducing the *S. aureus* biofilm; however, GAM is a much stronger antibiofilm compound than NGAM. Both compounds similarly reduce the expression of the *ica* genes responsible for the synthesis of PIA, which is critical for biofilm formation (Figure 8). GAM, compared to NGAM, facilitates the transition from a biofilm to a planktonic growth phase by reducing the expression of genes encoding initial adhesion and colonization, as well as those involved in arginine metabolism and TCA pathways. The strong anti-biofilm potency of GAM makes it a highly promising compound that could be used alone or in combination with other antimicrobial agents to reduce or prevent *S. aureus* biofilms in the future.

## Data Availability

The datasets presented in this study can be found in online repositories. The names of the repository/repositories and accession number(s) can be found in the article/Supplementary material.
