# Supplementary figures and images for: Identification of metabolic pathways modulated by GAM and NGAM in the inhibition of Staphylococcus aureus biofilm formation
Source: Front Microbiol. 2025 Nov 6;16:1689343. doi: 10.3389/fmicb.2025.1689343 (PMC12631271; doi:10.3389/fmicb.2025.1689343)

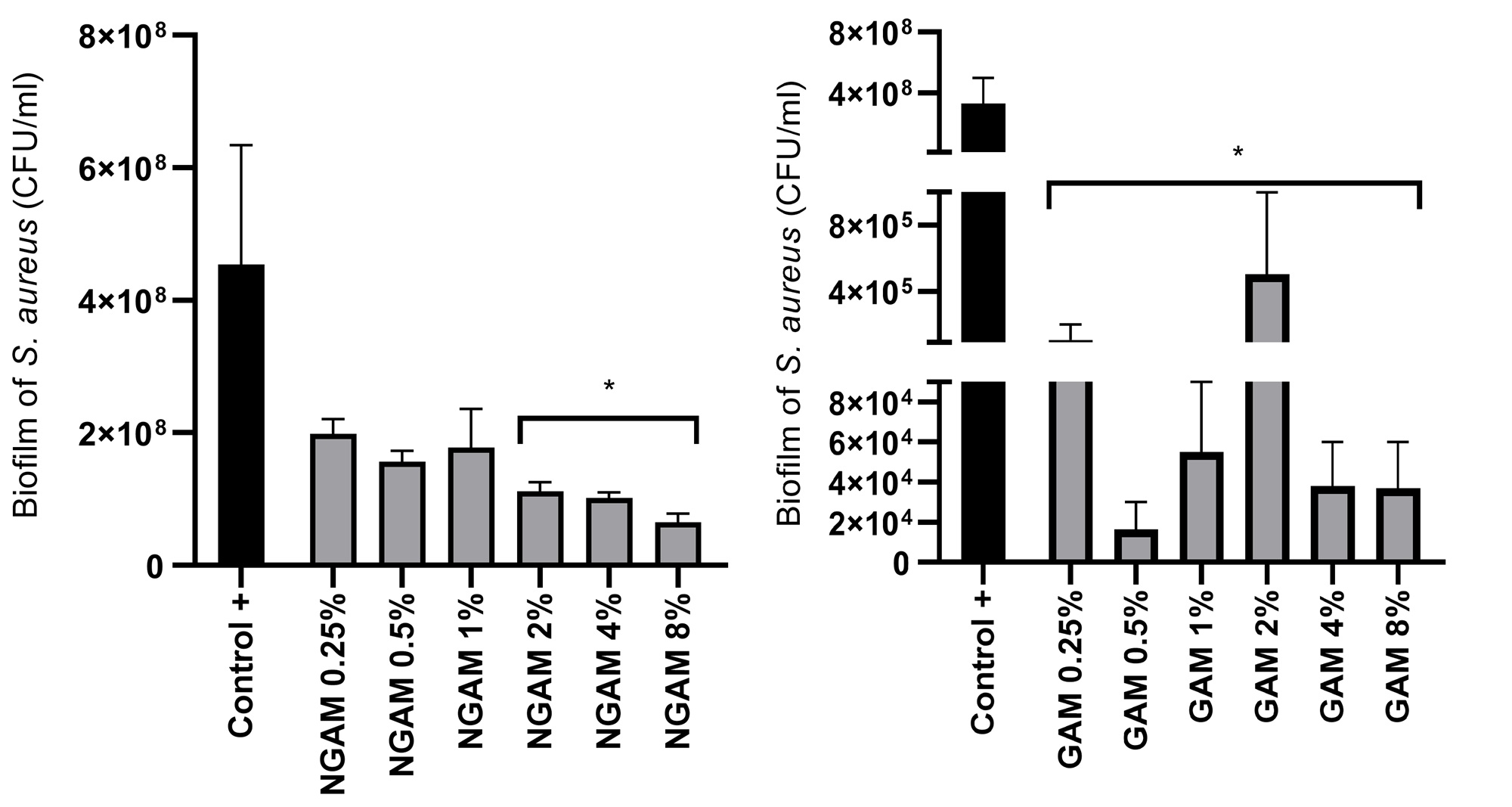

Supplement: Supplementary file 1 [file Image_1.JPEG]
